# Increased RTN3 phenocopies nonalcoholic fatty liver disease by inhibiting the AMPK–IDH2 pathway

**DOI:** 10.1002/mco2.226

**Published:** 2023-03-14

**Authors:** Hao Huang, Shuai Guo, Ya‐Qin Chen, Yu‐Xing Liu, Jie‐Yuan Jin, Yun Liang, Liang‐Liang Fan, Rong Xiang

**Affiliations:** ^1^ Department of Nephrology Xiangya Hospital Central South University Changsha China; ^2^ Department of Cell Biology School of Life Sciences Central South University Changsha China; ^3^ Hunan Key Laboratory of Animal Models for Human Diseases School of Life Sciences Central South University Changsha China; ^4^ National Clinical Research Center for Geriatric Disorders Xiangya Hospital Central South University Changsha China; ^5^ Department of Cardiology Second Xiangya Hospital Central South University Changsha China

**Keywords:** AMPK, IDH2, mitochondrial dysfunction, nonalcoholic fatty liver disease, RTN3

## Abstract

Reticulon 3 (RTN3), an endoplasmic reticulum protein, is crucial in neurodegenerative and kidney diseases. However, the role of RTN3 in liver tissues has not been described. Here, we employed public datasets, patients, and several animal models to explore the role of RTN3 in nonalcoholic fatty liver disease (NAFLD). The underlying mechanisms were studied in primary hepatocytes and L02 cells in vitro. We found an increased expression of RTN3 in NAFLD patients, high‐fat diet mice, and oxidized low‐density lipoprotein‐treated L02 cells. The RTN3 transgenic mice exhibited the phenotypes of fatty liver and lipid accumulation. Single‐cell RNA sequencing analysis indicated that increased RTN3 might induce mitochondrial dysfunction. We further showed this in primary hepatocytes, the L02 cell line, and the *Caenorhabditis elegans* strain. Mechanistically, RTN3 regulated these events through its interactions with glucose‐regulated protein 78 (GRP78), which further inhibited the adenosine 5 monophosphate‐activated protein kinase (AMPK)–isocitrate dehydrogenase 2 (IDH2) pathway. In the end, knockout of RTN3 relieved fatty liver and mitochondrial dysfunction. Our study indicated that RTN3 was important in NAFLD and lipid catabolism and that an increase in RTN3 in the liver might be a risk factor for nonalcoholic steatohepatitis and NAFLD.

## Graphical abstract

This study suggested that RTN3 can interact with GRP78 on the endoplasmic reticulum. The increased expression of RTN3 can facilitate interactions between RTN3 and GRP78, thereby decreasing the activity of GRP78 in regulating AMPK phosphorylation, which further reduces the expression IDH2, finally resulting in mitochondrial dysfunction, increased reactive oxygen species, and NAFLD.

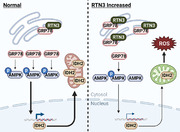

## 1 INTRODUCTION

Nonalcoholic fatty liver disease (NAFLD) is one of the most common hepatic diseases and is defined as the accumulation of fat in the liver induced by nonalcoholic mechanisms.^1^ It represents a clinical spectrum ranging from simple steatosis and nonalcoholic steatohepatitis (NASH) to cirrhosis and hepatocellular carcinoma.^2^ Currently, the estimated worldwide prevalence of NAFLD is approximately 25%, which has become a crucial contributor to extrahepatic chronic diseases such as atherosclerosis and coronary heart disease.^3^

As important organelles for lipid anabolism and catabolism, the endoplasmic reticulum (ER) and mitochondrion are crucial in the development and progression of NAFLD.^4^ Many molecules and pathways involved in ER stress and mitochondrial dysfunction have been reported to promote the genesis and development of NAFLD.^5^, ^6^ The discovery of new molecules that affect NAFLD will help us better understand the pathogenesis of NAFLD and aid in the treatment of this disease.^7^

Reticulon 3 (RTN3) is an ER protein belonging to the RTN family with a signature C‐terminal RTN homolog domain that is important in shaping the tubule ER structure.^8^ Functionally, RTN3 has been found to regulate the activity of Alzheimer's β‐secretase, mediate membrane contact between the ER and plasma membrane by interacting with the cytosolic region of epidermal growth factor receptor, promote lipid synthesis by activating sterol regulatory element‐binding protein 1c (SREBP1c) in adipose tissue, and induce chronic kidney disease via the insulin‐like growth factor 2 (IGF2) pathway.^9^, ^10^, ^11^, ^12^ However, the role of RTN3 in liver tissues has not been described.

This study showed a strong correlation between increased RTN3 levels and NAFLD. Both transgenic mouse models overexpressing the wild‐type (WT) human RTN3 gene (Tg‐RTN3) and *Caenorhabditis elegans* strain overexpressing the RTN3 homology gene (Tg‐Ret1) presented with overt NAFLD and lipid accumulation. A mechanistic study revealed that the overexpression of RTN3 inhibited the adenosine 5 monophosphate‐activated protein kinase (AMPK)–isocitrate dehydrogenase 2 (IDH2) pathway by altering the interaction between RTN3 and glucose‐regulated protein 78 (GRP78), inducing mitochondrial dysfunction, which ultimately promotes the genesis and development of NAFLD. Finally, we found that reducing the expression of RTN3 can rescue the NAFLD and mitochondrial dysfunction caused by a high‐fat diet (HFD) by activating the AMPK–IDH2 pathway. Hence, our study indicated that overexpression of RTN3 in the liver might be a risk factor for NAFLD, and RTN3 may be a potential therapeutic target for NAFLD.

## 2 RESULTS

### 2.1 Link between high expression of RTN3 and NAFLD

**FIGURE 1:**
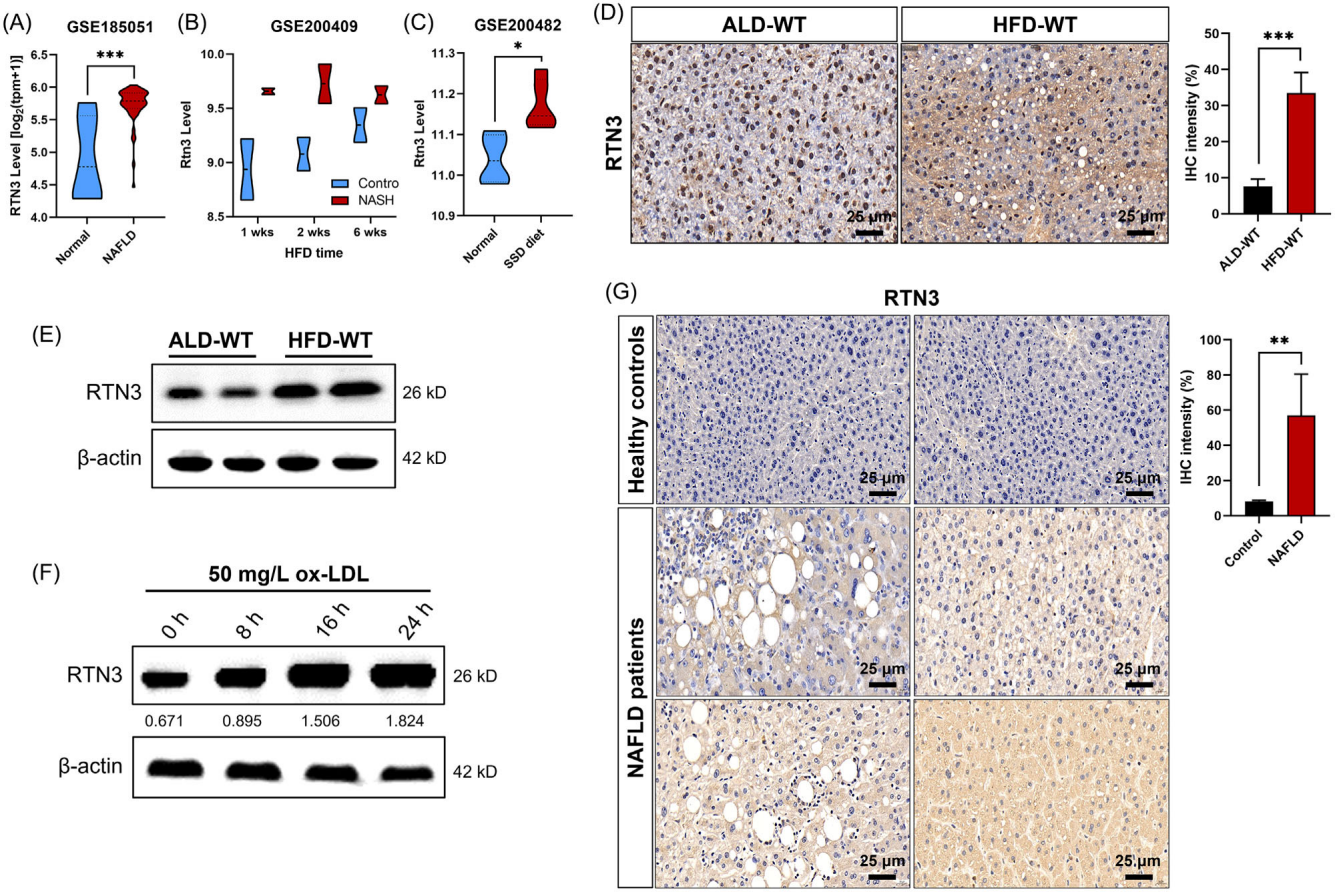
Link between high expression of reticulon 3 (RTN3) and nonalcoholic fatty liver disease (NAFLD). The mRNA levels of RTN3 in NAFLD patients and healthy control (A), in high‐fat diet (HFD) mice and wild‐type (WT) control (B), and in steatosis‐steatohepatitis diet (SSD) mice and WT control (C). The protein levels of RTN3 were detected in ad libitum diet (ALD)‐WT and HFD‐WT mice liver tissues by immunohistochemistry (IHC) (D) and western blotting (WB) (E). (F) WB showed the RTN3 levels in oxidized low‐density lipoprotein (ox‐LDL) (50 mg/L) treated L02 cell lines in different time. (G) IHC showed the RTN3 levels in liver tissues of healthy controls and NAFLD patients. ^*^ *p* < 0.05, ^**^ *p* < 0.01, and ^***^ *p* < 0.001. NASH: nonalcoholic steatohepatitis.

Because no previous study focused on RTN3 and liver diseases, we first analyzed the RNA levels of RTN3 in public datasets (GSE185051, GSE200409, and GSE200482). The results showed that the RNA levels of RTN3 were increased dramatically in the NAFLD patient group, HFD mouse group, and steatosis‐steatohepatitis diet (SSD) mouse group compared to healthy controls (Figure 1A–C). We then generated the NAFLD mouse model by HFD feeding. The immunohistochemistry (IHC) and western blot (WB) analysis revealed that the protein levels of RTN3 were greater in HFD‐WT mouse liver tissues (*n* = 6) than in the ad libitum diet (ALD‐WT) group (*n* = 6) (Figure 1D,E). According to previous study in NAFLD,^13^ we selected normal human liver cell line L02 to perform the in vitro experiment. We treated L02 cells with oxidized low‐density lipoprotein (ox‐LDL). WB analysis showed that the longer the time treated with ox‐LDL, the higher the expression of RTN3 was (Figure 1F). Finally, we collected liver tissues from four NAFLD patients and two healthy controls (liver contusion patients) (Table S1). IHC analysis exhibited that the protein levels of RTN3 in NAFLD patients were greater than those in healthy controls (Figure 1G). These findings in public databases, patients, mice, and cell lines indicated a strong correlation between increased RTN3 levels and NAFLD.

### 2.2 Overexpression of RTN3 exhibits NAFLD and fat accumulation

**FIGURE 2:**
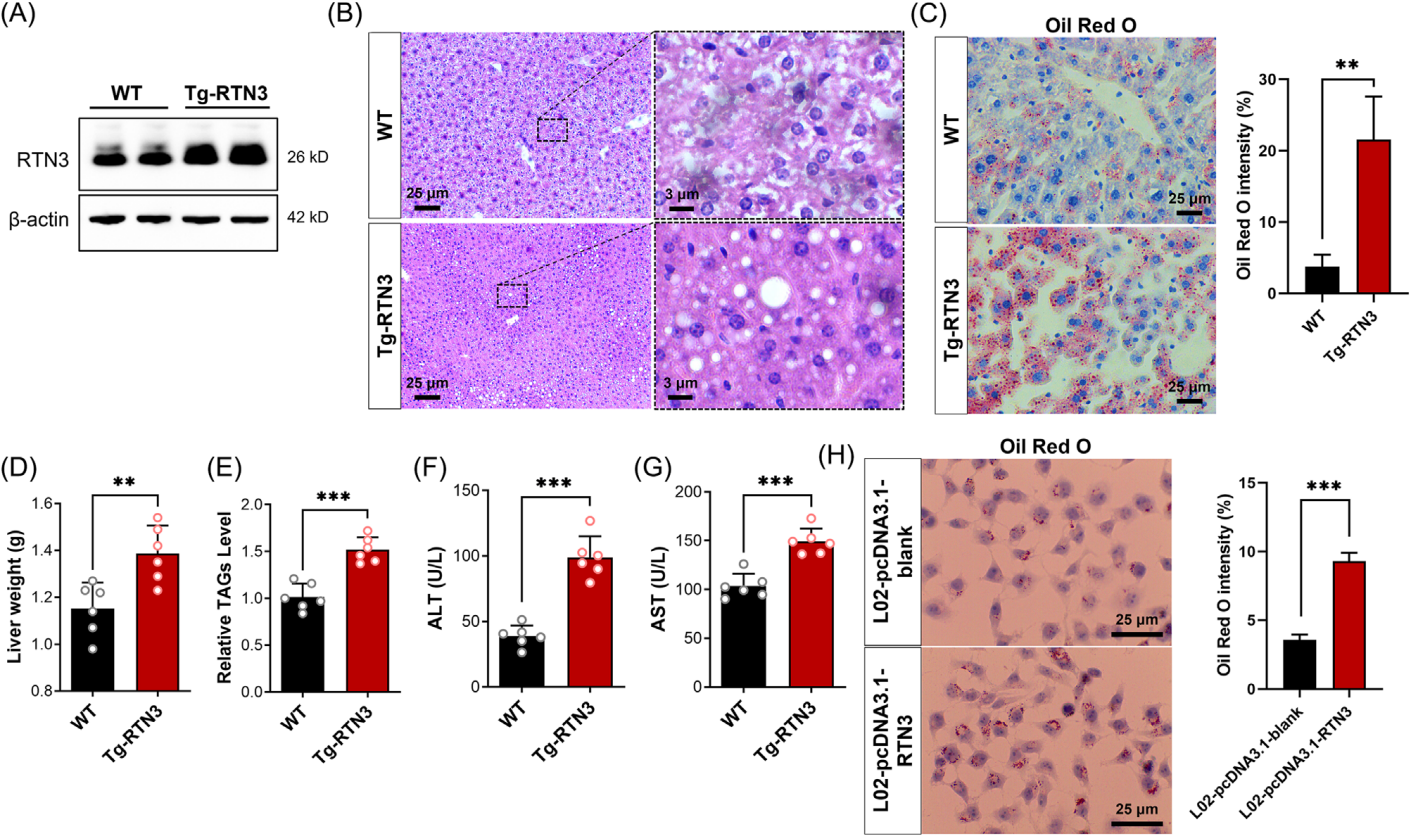
Overexpression of reticulon 3 (RTN3) exhibits nonalcoholic fatty liver disease (NAFLD) and fat accumulation. (A) Western blotting (WB) detected the RTN3 protein levels in wild‐type (WT) and Tg‐RTN3 mice liver tissues. (B) Hematoxylin–eosin (HE) staining and (C) Oil red O staining of WT and Tg‐RTN3 mice liver tissues. Liver weight (D) and liver triglyceride (TAG) levels (E) of WT and Tg‐RTN3 mice. The alanine aminotransferase (ALT) (F) and aspartate aminotransferase (AST) (G) levels in WT and Tg‐RTN3 mice serum. (H) Oil red O staining of L02 cells transfected with pcDNA3.1‐blank or pcDNA3.1‐RTN3. ^**^ *p* < 0.01 and ^***^ *p* < 0.001.

To reveal the relationship between RTN3 and NAFLD, we generated Tg‐RTN3 mice (Figure 2A). Hematoxylin–eosin (HE) staining and Oil Red O staining indicated that the Tg‐RTN3 mice (*n* = 6) presented with overt fat accumulation at 4 months of age with standard chow (*n* = 6) (Figure 2B,C). The liver weight and triglyceride (TAG) levels in liver tissues of Tg‐RTN3 mice were approximately 20.39% and 48.02% more than those in WT littermates (Figure 2D,E). Simultaneously, the liver function tests showed that the levels of serum alanine aminotransferase (ALT) and aspartate aminotransferase (AST) in Tg‐RTN3 mice were also much greater than those in their WT littermates (Figure 2F,G). In addition, the L02 cells transfected with pcDNA3.1‐RTN3 also presented more lipid accumulation than cells transfected with pcDNA3.1‐blank (Figure 2H). These observations suggested that overexpression of RTN3 may lead to NAFLD and fat accumulation.

### 2.3 Increased RTN3 is associated with lipid oxidation and mitochondrial respiration in NASH mice

**FIGURE 3:**
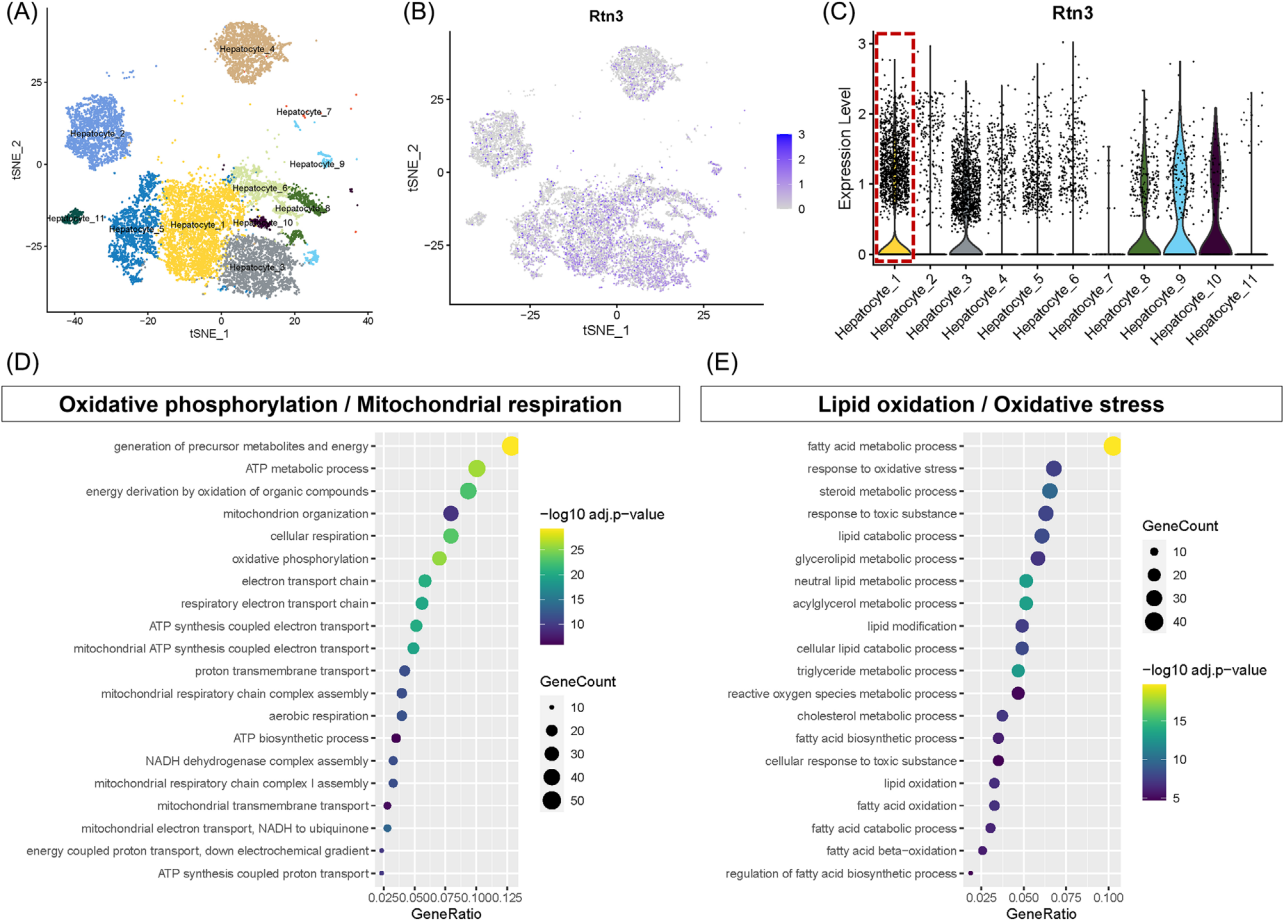
Increased reticulon 3 (RTN3) is associated with lipid oxidation and mitochondrial respiration from the nonalcoholic steatohepatitis (NASH) mice. (A) Composition and distribution of single cells from GSE166504. (B) The distribution profile of RTN3 for each cell by the t‐distributed stochastic neighbor embedding (t‐SNE) plot. (C) The violin plots of the expression profile of RTN3 in GSE166504. Dot plots summarizing the (D) upregulated and (E) downregulated enriched Gene Ontology (GO) in Rtn3 high expressed hepatocyte_1 cells. Dots are colored by the q‐value of significantly upregulated or downregulated genes within each gene set (<0.05 false discovery rate (FDR)).

Next, to further identify RTN3‐expressing hepatocyte subpopulations, a supervised analysis was performed on a previously published single‐cell RNA sequencing (scRNA‐seq) dataset isolated from the live tissues of chow diet, 15‐ and 30‐week high‐fat high‐fructose diet (HFHFD) mice, in which hepatocyte subpopulations unique to NASH were reported.^14^ According to previous research, 11 hepatocyte subpopulations were identified in NASH model mice (Figure 3A). The results revealed the differential distribution of RTN3 among different NASH hepatocyte clusters (Figure 3B). RTN3 was predominantly highly expressed in the hepatocyte_1 cluster (Figure 3C). Analysis of Gene Ontology (GO) enrichment in cluster hepatocyte_1 suggested increased TAG and acylglycerol metabolic processes, as well as decreased adenosine triphosphate (ATP) metabolic processes, cellular respiration, and oxidative phosphorylation, in hepatocytes with high RTN3 expression (Figure 3D,E). Together, our results represent a comprehensive characterization of RTN3 in NASH, and the increased RTN3 showed potential functional importance in lipid oxidation and mitochondrial respiration.

### 2.4 Increased RTN3 can disrupt the morphology and function of mitochondria

**FIGURE 4:**
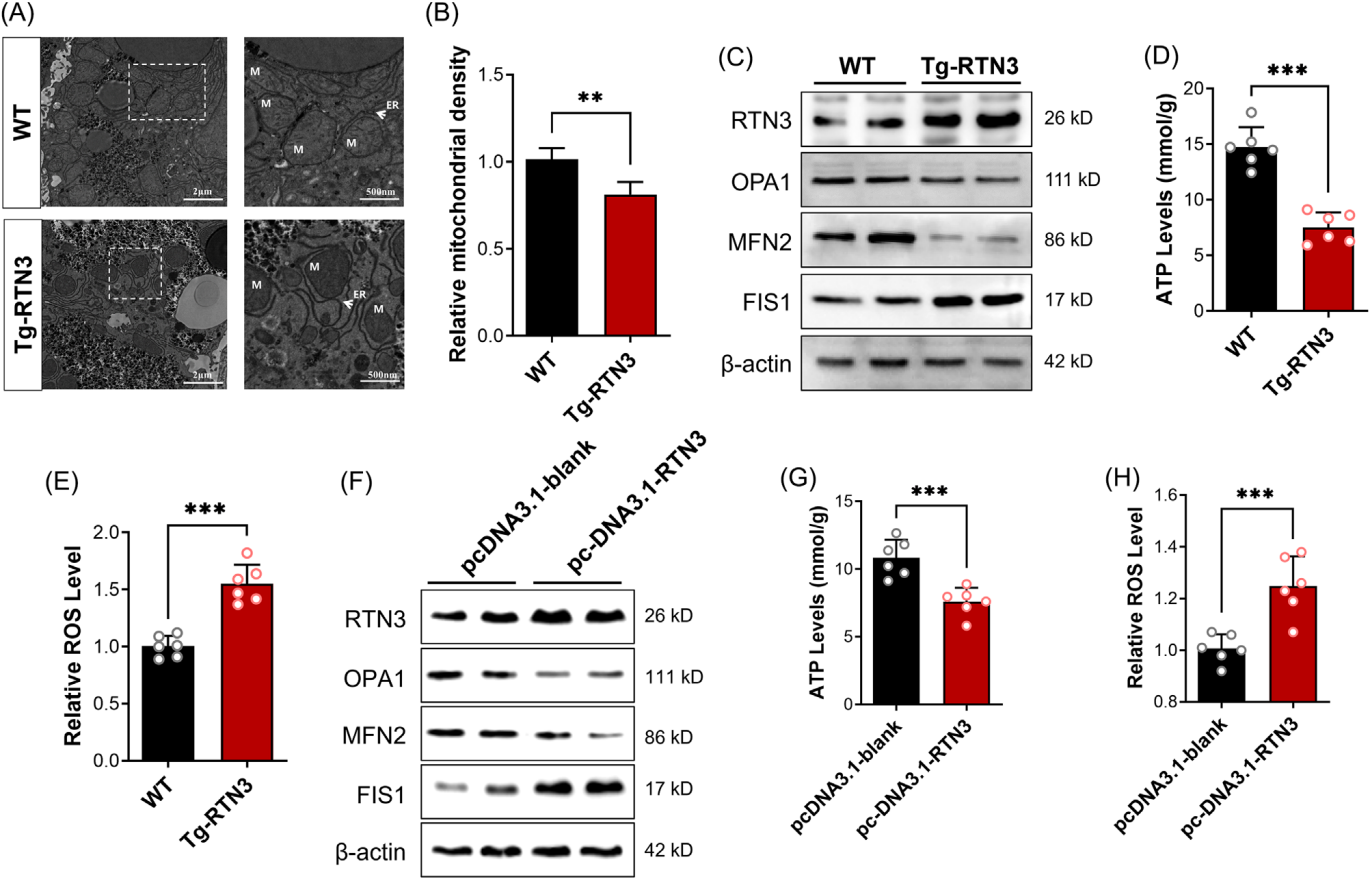
Increased reticulon 3 (RTN3) can disrupt the morphology and function of mitochondria. (A) Transmission electron microscopy (TEM) described the mitochondria condition in liver tissues of wild‐type (WT) and Tg‐RTN3 mice. ER, endoplasmic reticulum; M, mitochondria. (B) The statistical results of the relative mitochondrial density. (C) The expression of OPA1, mitofusin 2 (MFN2), and fission mitochondrial 1 (FIS1) in primary hepatocytes from RTN3‐Tg mice and WT mice. The levels of adenosine triphosphate (ATP) (D) and reactive oxygen species (ROS) (E) in primary hepatocytes from RTN3‐Tg mice and WT mice. (F) The expression of OPA1, MFN2, and FIS01 in L02 cells transfected with pcDNA3.1‐blank or pcDNA3.1‐RTN3. The levels of ATP (G) and ROS (H) in L02 cells transfected with pcDNA3.1‐blank or pcDNA3.1‐RTN3. ^**^ *p* < 0.01 and ^***^ *p* < 0.001.

Because scRNA‐seq indicated that increased RTN3 was related to mitochondrial respiration in NASH mouse models, we detected the morphology and function of mitochondria in the hepatocytes of Tg‐RTN3 mice. Transmission electron microscopy (TEM) exhibited that the size and density of hepatocyte mitochondria were significantly decreased in Tg‐RTN3 mice compared to controls (Figure 4A,B). Moreover, a loss of mitochondrial cristae structure was observed in the hepatocytes of Tg‐RTN3 mice (Figure 4A). Mitofusin 2 (MFN2) and fission mitochondrial 1 (FIS1) are responsible for regulating the fusion and fission of mitochondria, and optic atrophy 1 (OPA1) mitochondrial dynamin‐like GTPase (OPA1) plays a crucial role in maintaining the structure and function of mitochondrial cristae.^15^ We then performed WB analysis of primary hepatocytes separated from Tg‐RTN3 and WT mice. The results showed reduced protein levels of MFN2 and OPA1 and increased protein levels of FIS1 in Tg‐RTN3 hepatocytes (Figure 4C), which indicated that overexpression of RTN3 may promote the fission of mitochondria and inhibit the fusion of mitochondria. This result was consistent with our TEM observations that in Tg‐RTN3 mice liver tissues, the size of mitochondria was reduced, and mitochondrial cristae structure was lost. Further detection of the levels of ATP and mitochondrial reactive oxygen species (ROS) showed that compared to those in WT hepatocytes, the ATP levels in Tg‐RTN3 hepatocytes were dramatically reduced (Figure 4D), and the ROS levels were increased (Figure 4E). We then transfected the pcDNA3.1‐RTN3 plasmid into L02 cells and detected a similar tendency in the levels of proteins that control mitochondrial morphology, as well as ATP and ROS levels (Figure 4F–H).

**FIGURE 5:**
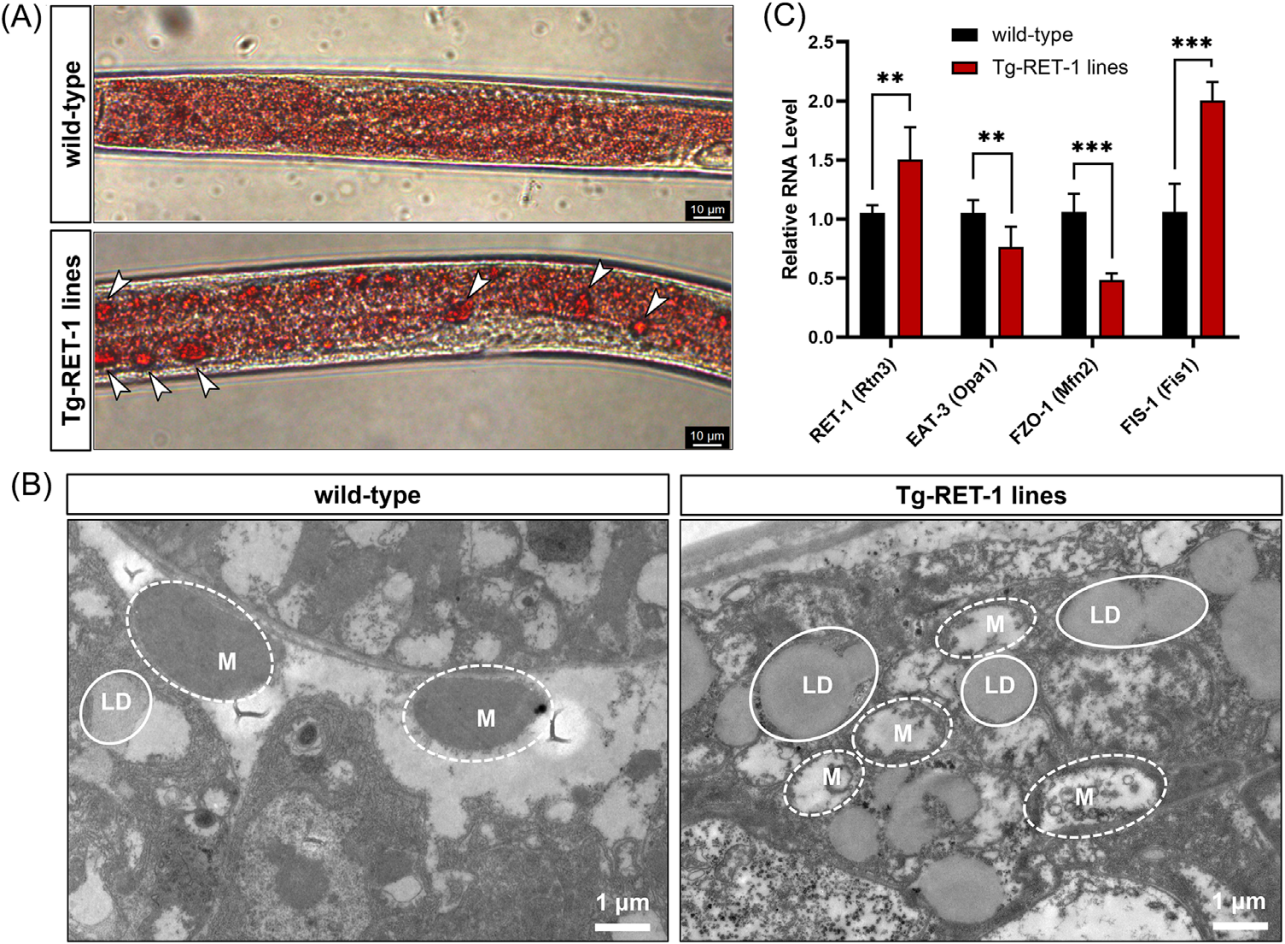
Increased Ret1 can lead to lipid accumulation and mitochondrial dysfunction in *Caenorhabditis elegans*. (A) Oli red O staining of the wild‐type (WT) and Tg‐Ret1 *C. elegans*. The arrows indicated the large lipid droplets. (B) Transmission electron microscopy (TEM) describing the mitochondria and lipid droplets condition of the WT and Tg‐Ret1 *C. elegans*. LD, lipid droplet; M, mitochondria. (C) Real‐time polymerase chain reaction (RT‐PCR) revealed the mRNA levels of Ret1, EAT3, FZO1, and fission mitochondrial 1 (FIS1) in WT and Tg‐Ret1 *C. elegans*. ^**^ *p* < 0.01 and ^***^ *p* < 0.001.

Ret1 is a homologous gene of RTN3 in *C. elegans*. In our generated Tg‐Ret1 *C. elegans*, Oil red O staining indicated that the number of large lipid drops in Tg‐Ret1 was much greater than that in the WT (Figure 5A). The TEM of Tg‐Ret1 *C. elegans* displayed fragmented mitochondria and increased lipid droplets compared to WT (Figure 5B). Real‐time polymerase chain reaction (RT‐PCR) also suggested that the mRNA levels in the C. elegans Mfn1,2 homologue (FZO1) (Mfn2) and the C. elegans Opa1 homologue (EAT3) (Opa1) were decreased in Tg‐Ret1 *C. elegans* compared to WT, while the expression of mRNA in Fis1 was increased (Figure 5C).

These discoveries in mouse hepatocytes and *C. elegans* confirmed that overexpression of RTN3 can disrupt the morphology and function of mitochondria, which may further affect lipid catabolism in hepatocytes and result in NAFLD.

### 2.5 Increased RTN3 can inhibit the AMPK–IDH2 pathway by interacting with GRP78

**FIGURE 6:**
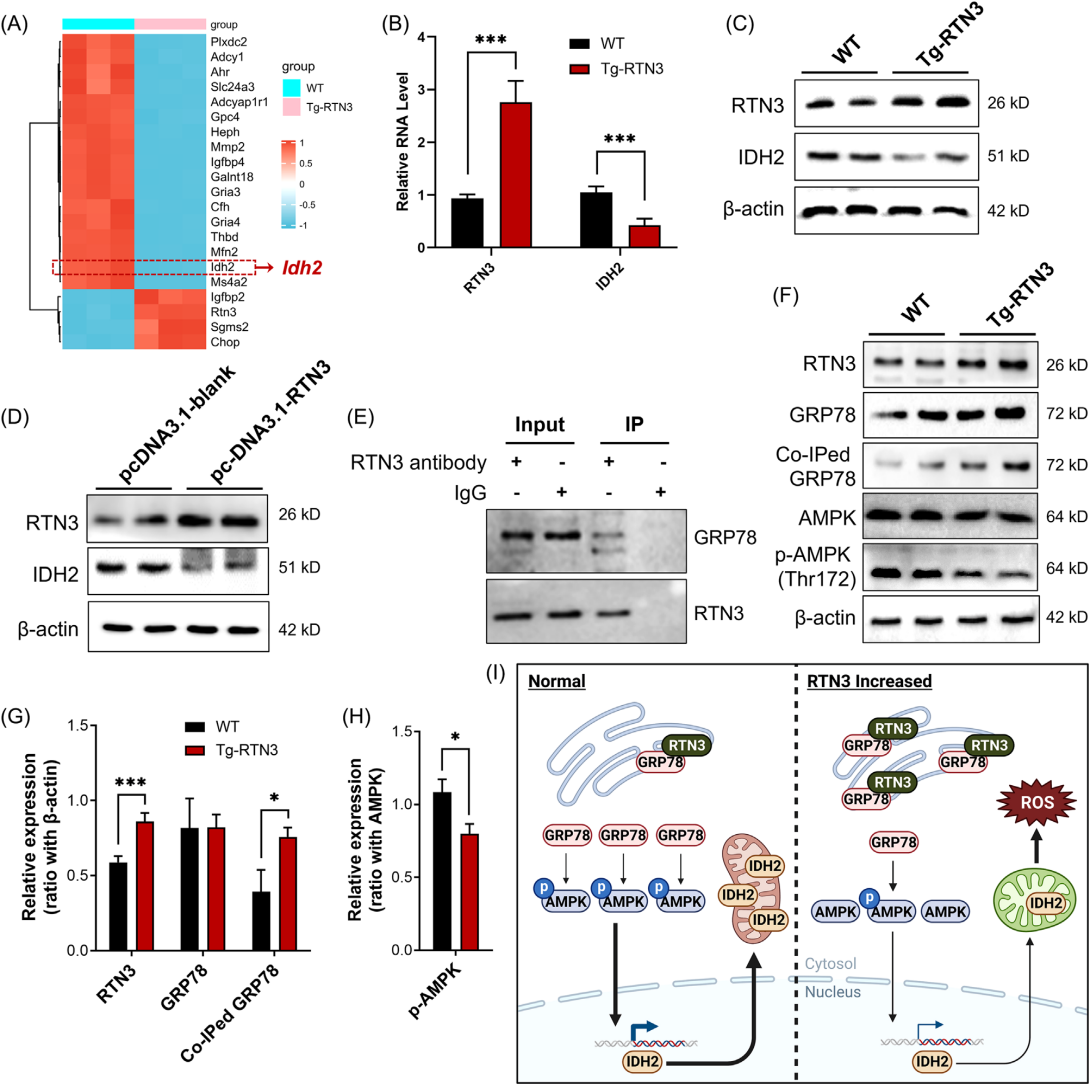
Increased reticulon 3 (RTN3) can inhibit adenosine 5 monophosphate‐activated protein kinase (AMPK)–isocitrate dehydrogenase 2 (IDH2) pathway via interacting with glucose‐regulated protein 78 (GRP78). (A) Significantly differentially expressed genes between wild‐type (WT) and Tg‐RTN3 mice liver tissues revealed by RNA‐seq data. (B) Real‐time polymerase chain reaction (RT‐PCR) showed the mRNA levels of RTN3 and IDH2 in WT and Tg‐RTN3 mice liver tissues. Western blotting (WB) analyzed the IDH2 levels in (C) WT and Tg‐RTN3 mice liver tissue group, and in (D) L02 cell lines transfected with pcDNA3.1‐blank and pcDNA3.1‐RTN3 group. (E) Coimmunoprecipitation (Co‐IP) confirmed that RTN3 can interact with GRP78 in mouse liver tissues. (F) WB and Co‐IP analysis showing the levels of GRP78, coimmunoprecipitated GRP78, AMPK, and p‐AMPK in primary hepatocytes from RTN3‐Tg mice and WT mice. (G) The statistical results of the RTN3, GRP78, and Co‐IPed GRP78. (H) The statistical results of the p‐AMPK. (I) Potential mechanism of how higher RTN3 expression induces mitochondrial dysfunction in liver. The figure was created with BioRender.com. ^*^ *p* < 0.05 and ^***^ *p* < 0.001.

We then employed RNA‐seq to investigate the potential pathways between increased RTN3 and mitochondrial dysfunction. We found that a crucial gene, IDH2, was changed dramatically in the liver tissues of Tg‐RTN3 mice compared to WT littermates at 4 months of age fed standard chow (Figure 6A). IDH2, an enzyme located in the inner membrane of mitochondria, belongs to the family of isocitrate dehydrogenases (IDHs), which are significant in regulating intermediary metabolism and energy production.^16^, ^17^ Former studies have indicated that a reduction in IDH2 can lead to mitochondrial dysfunction and NAFLD.^18^, ^19^ RT‐PCR and WB also confirmed the reduction in IDH2 in Tg‐RTN3 primary hepatocytes or L02 cells transfected with pcDNA‐RTN3 compared to WT controls (Figure 6B–D).

However, it is unclear how RTN3 regulates the expression of IDH2 and ultimately leads to mitochondrial dysfunction and NAFLD. Mass spectrometry (MS) was used to detect the candidate RTN3‐interacting molecules in WT liver tissues. Interestingly, GRP78 was found to be a possible RTN3‐interacting protein (data not shown). Coimmunoprecipitation (Co‐IP) analysis further validated the interaction between RTN3 and GRP78 in primary hepatocytes (Figure 6E). GRP78, also known as binding immunoglobulin protein (BiP), is an ER protein that facilitates a wide range of protein folding processes.^20^ Simultaneously, GRP78 has been proved to regulate the expression of AMPK,^21^ a guardian of metabolism and mitochondrial homeostasis,^22^ which has been proved can regulate the transcription of IDH2.^23^, ^24^, ^25^ Further WB and Co‐IP studies also suggested that the protein levels of GRP78 were not changed by increasing the RTN3 levels. However, the amount of coimmunoprecipitated GRP78 was much higher in Tg‐RTN3 group than in WT group, and this increased interaction appeared to inhibit activated AMPK (p‐AMPK) (Figure 6F–H).

Collectively, our data suggested that RTN3 can interact with GRP78 on the ER. The overexpression of RTN3 can promote the interactions between RTN3 and GRP78, thereby reducing the ability of GRP78 in regulating AMPK phosphorylation, which further reduces the expression IDH2, finally resulting in mitochondrial dysfunction, increased ROS, and NAFLD (Figure 6I).

### 2.6 Decreased RTN3 can rescue NAFLD and mitochondrial dysfunction caused by HFD by activating the AMPK–IDH2 pathway

**FIGURE 7:**
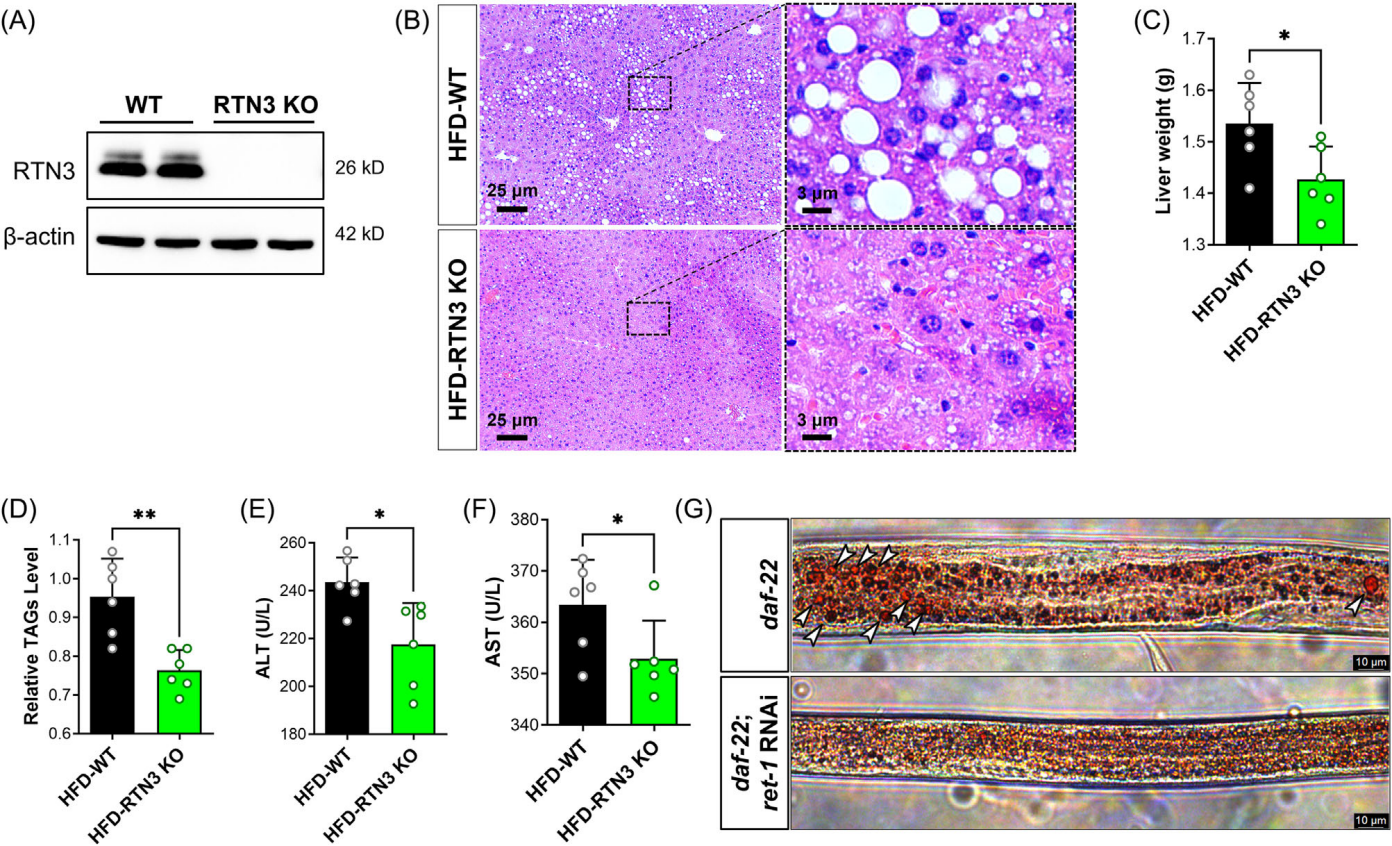
Decreased reticulon 3 (RTN3) can rescue nonalcoholic fatty liver disease (NAFLD) caused by high‐fat diet (HFD). (A) Western blotting (WB) detected the RTN3 protein levels in wild‐type (WT) and RTN3 knockout (KO) mice liver tissues. (B) Hematoxylin–eosin (HE) staining of high‐fat diet (HFD)‐WT and HFD‐RTN3 KO mice liver tissues. Liver weight (C) and liver triglyceride (TAG) levels (D) of HFD‐WT and HFD‐RTN3 KO mice. The alanine aminotransferase (ALT) (E) and aspartate aminotransferase (AST) (F) levels in HFD‐WT and HFD‐RTN3 KO mice serum. (G) Oil red O staining of daf‐22 and daf‐22: RNAi *Caenorhabditis elegans*. The arrows indicated the large lipid droplets. ^*^ *p* < 0.05 and ^**^ *p* < 0.01.

**FIGURE 8:**
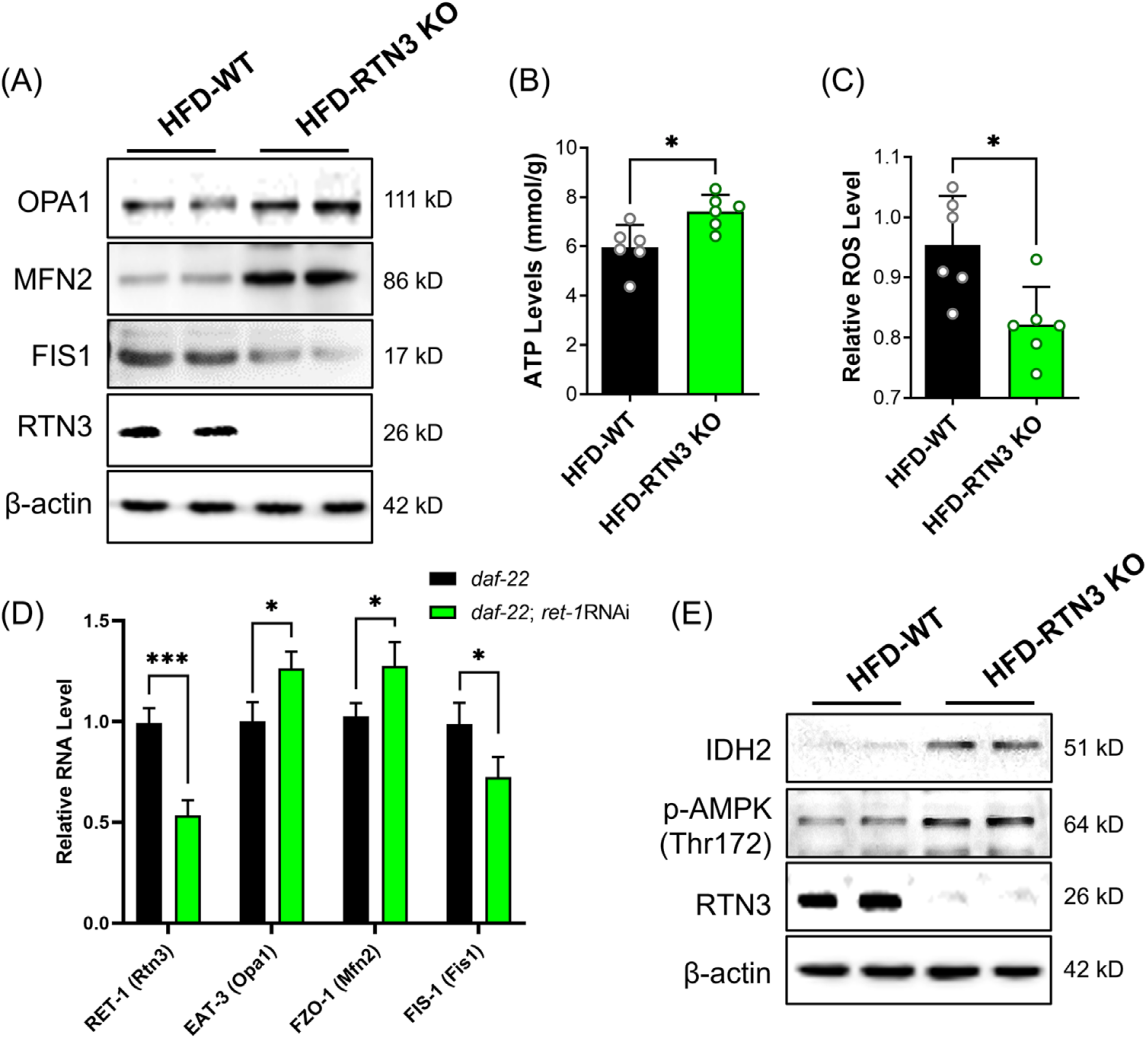
Decreased reticulon 3 (RTN3) can rescue mitochondrial dysfunction caused by high‐fat diet (HFD) via activating adenosine 5 monophosphate‐activated protein kinase (AMPK)–isocitrate dehydrogenase 2 (IDH2) pathway. (A) The expression of OPA1, mitofusin 2 (MFN2), and fission mitochondrial 1 (FIS1) in liver tissues from HFD‐WT mice and HFD‐RTN3 knockout (KO) mice. The levels of adenosine triphosphate (ATP) (B) and reactive oxygen species (ROS) (C) in liver tissues from HFD‐WT mice and HFD‐RTN3 KO mice. (D) Real‐time polymerase chain reaction (RT‐PCR) revealed the mRNA levels of Ret1, EAT3, FZO1, and FIS1 in dat‐22 and daf‐22: RNAi *Caenorhabditis elegans*. (E) The expression of IDH2 and p‐AMPK in liver tissues from HFD‐WT mice and HFD‐RTN3 KO mice. ^*^ *p* < 0.05.

RTN3‐null (RTN3 knockout [KO]) mice were also generated to further confirm the relationship between RTN3 and NAFLD (Figure 7A). We found that RTN3 KO mice (*n* = 6) exhibited less fat accumulation in liver tissues than WT mice (*n* = 6) after 3 months of HFD feeding (Figure 7B). The liver weight and TAG levels in the HFD‐RTN3 KO group were lower than those in the HFD‐WT group (Figure 7C,D). Liver function tests also suggested that the levels of ALT and AST were reduced in HFD‐RTN3 KO mice compared with HFD‐WT mice (Figure 7E,F). In addition, we knocked down the expression of Ret1 in obese worms (daf‐22) by RNAi and found that the number of larger lipid drops was reduced in the daf‐22; Ret1 RNAi group compared to the daf‐22 group (Figure 7G). All these observations suggest that reducing the expression of RTN3 can relieve the NAFLD and lipid accumulation caused by HFD. Referring to the aforementioned mechanism, we also detected the expression of OPA1, MFN2, and OPA1, and levels of ATP and ROS in the liver tissues of HFD‐RTN3 KO mice. The data exhibited that compared to HFD‐WT group, the protein levels of MFN2 and OPA1, and the ATP levels were increased, while the protein levels of FIS1 and ROS levels were decreased in HFD‐RTN3 KO group (Figure 8A–C). Similar results were also detected in daf‐22; Ret1 RNAi *C. elegans* compared to daf‐22 *C. elegans* by RT‐PCR (Figure 8D). Finally, an elevatory tendency of IDH2 and p‐AMPK was detected by WB in HFD‐RTN3 KO mice liver tissues (Figure 8E).

In RTN3 KO mice and related *C. elegans*, studies have indicated that reducing the expression of RTN3 can rescue lipid accumulation, liver function, and mitochondrial dysfunction caused by HFD by activating the AMPK–IDH2 pathway. Reducing the expression of RTN3 in the liver may be a potential therapeutic strategy for treating NAFLD.

## 3 DISCUSSION

As an ER membrane protein, RTN3 plays a significant role in the nervous system.^26^, ^27^ Although richly expressed in neurons, this protein is expressed in many tissues and has eight spliced forms, and only a few studies have shown the role and function of RTN3 in peripheral tissues.^11^, ^12^ In mammals, RTN3 and reticulon 4 (RTN4) are the only two RTN family members expressed in the liver.^28^ Previous studies have demonstrated that RTN4 can facilitate hepatocyte proliferation and liver regeneration, suggesting that RTN4 is an important regulator of hepatic fibrosis.^29^, ^30^ Moreover, the RTN4 receptor can activate the protein kinase alpha pathway and increase the nuclear translocation of liver X receptor α, promoting hepatic lipogenesis.^31^ However, as the homolog of RTN4, the function of RTN3 in liver tissues was not previously investigated. Here, we provided the evidence that the increased expression of hepatic RTN3 leads to fat accumulation and NAFLD due to mitochondrial dysfunction and increased ROS. This conclusion was supported by experiments in mice, *C. elegans*, cell lines, and NAFLD patient samples, which help us understand how RTN3 and its interacting proteins contribute to mitochondrial dysfunction and NAFLD. Drug development for related targets such as RTN3 may be a potential therapeutic strategy for treating NAFLD.^7^

In our study, we revealed that increased RTN3 expression may lead to mitochondrial dysfunction in hepatocytes. Previous studies reported that structural and functional alterations of mitochondria in hepatocytes contribute to the pathogenesis of NAFLD.^32^, ^33^ Mitochondrial dysfunction impairs the ability to handle increased lipid flux, thereby disrupting lipid catabolism; respiratory oxidation may collapse with impairment of fat homeostasis, generation of lipid‐derived toxic metabolites, and overproduction of ROS, which further contribute to NAFLD.^34^, ^35^ In this study, we first report that increased RTN3 may induce mitochondrial dysfunction and that the mitochondrial regulatory role of RTN3 is evolutionarily conserved.

By employing biochemical approaches, we identified overtly decreased expression of IDH2 in the liver tissues of Tg‐RTN3 mice. Previous studies have demonstrated that a reduction in IDH2, an enzyme located in mitochondria, may lead to mitochondrial dysfunction in hepatocytes and cardiomyocytes.^16^, ^17^, ^36^ Simultaneously, the latest studies have indicated that IDH2‐null mice exhibit mitochondrial damage, increased ROS, and hepatic lipid dysregulation.^18^ Previous studies have shown that increased ROS can directly promote lipid deposition in the liver, which is the pathogenesis of NAFLD.^34^ IDH2 is an enzyme located at the inner mitochondrial membrane, while RTN3 is a tubular ER protein. How RTN3 impacts IDH2 expression is an important question. Here, we showed that RTN3 can interact with GRP78, which can further regulate the AMPK pathway, the upstream signaling pathway of IDH2.^21^, ^23^, ^24^, ^37^ Previous studies have suggested that AMPK is the key molecule in regulating lipid metabolism by phosphorylation. GRP78 can activate AMPK to ameliorate dexamethasone‐induced fatty liver disease in C57BL/6 mice.^21^ Vaspin can attenuate steatosis‐induced fibrosis via the GRP78 receptor by targeting the AMPK signaling pathway. As the upstream molecule of IDH2, AMPKα has been proven to regulate IDH2 transcription.^23^, ^25^ AMPKα1 deficiency may attenuate IDH2 expression in mice, which is consistent with our studies in mice. Hence, increased RTN3 may bind more GRP78, which reduces the activity of GRP78 in regulating p‐AMPK, attenuates IDH2 expression, and induces mitochondrial dysfunction and NAFLD.

Recently, some studies have focused on the role of RTN3 in physiology and pathology. Mutations in RTN3 have been detected in early‐onset Alzheimer's disease.^38^ One study found that RTN3 may mediate checkpoint kinase 2 activation and suppress hepatocellular carcinogenesis.^39^ In lipid research, one study found that RTN3 can alter very low‐density lipoprotein secretion in HepG2 cells,^40^ but in our Tg‐RTN3 mice, we only found that TAG levels were increased.^11^ Formerly, we found that increased RTN3 may activate the SREBF chaperone (SCAP)–SREBP1 pathway by interacting with heat shock protein family A (Hsp70) member 5 in fat tissues. The expression of SREBP1 was dramatically increased in the fat tissues of Tg‐RTN3 mice, which proved that RTN3 is crucial in regulating lipid anabolism.^11^ In the RTN3 KO mouse model, we also found that deletion of RTN3 can activate the IGF2–Janus kinase 2 (JAK2) pathway, which may further lead to chronic kidney disease and renal fibrosis.^12^ Here, we found that increased RTN3 can also disrupt the structure and function of mitochondria. Since the disruption of mitochondria may promote the levels of ROS and have a crucial role in TAG catabolism in liver tissues,^41^, ^42^ our studies further confirmed the function of RTN3 in TAG catabolism in liver tissues via the AMPK–IDH2 pathway by employing multiple mouse and *C. elegans* models.

## 4 CONCLUSIONS

In summary, our study suggests that high expression of RTN3 is a possible lesion for lipid deposition in the liver by impairing mitochondria and increasing ROS levels, partly through competitive binding with GRP78 to attenuate the GRP78‐mediated AMPK–IDH2 pathway. Reducing the expression of RTN3 may be a potential therapeutic method for NAFLD. Our findings also imply that RTN3 is a pivotal upstream regulator of the AMPK–IDH2 pathway that functions in mitochondrial biological homeostasis.

## 5 MATERIALS AND METHODS

### 5.1 Human tissues

Human liver sections were collected from Xiangya Hospital and the Second Xiangya Hospital. This study was approved by the Institutional Review Board committee of Central South University in China (approval number: no. 2021‐1‐1 for human specimens, no. 2021‐2‐1 for animals, date: February 24, 2021).

### 5.2 Mouse strains, *C. elegans* strains, cell lines, and key reagents

Tg‐RTN3 and RTN3 KO mice were described previously.^11^ The WT mice (C57BL/6J) were purchased from Cyagen Company (SuZhou, China). Male mice at 7–8 weeks of age were selected to feed a HFD consisting of 60% fat, 20% protein, and 20% carbohydrate for 3 months.

The WT *C. elegans* (N2 strain), Tg‐Ret1 *C. elegans* strain, and Daf‐22 *C. elegans* strain were purchased or generated as previously described.^11^

Primary hepatocytes were isolated from mouse liver tissues as follows: fresh liver tissues were separated from newborn mice and cut up by scissors. The fragmented tissues were washed with phosphate‐buffered saline with 100 U/mL penicillin and 100 μg/mL streptomycin and digested with collagenase/hyaluronidase. After filtration and centrifugation, the separated cells were cultured with complete medium. The L02 cell line was purchased from the GuangZhou Jennio Biotech Co., Ltd. (GuangZhou, China) and cultured with complete medium. Both primary hepatocytes and the L02 cell line were maintained at 37°C in a humidified, 5% CO_2_‐controlled atmosphere (Thermo Fisher Scientific).

The RTN3 antibody was generated in the Yan laboratory.^26^ The other key reagents are presented in Table S2.

### 5.3 Immunohistochemistry

Tissues were fixed with formalin and embedded in paraffin. Six‐micrometer sections were prepared for baking and dewaxing. After antigen retrieval and blocking, the RTN3 antibody was incubated for 8 h. Then, a broad spectrum IHC kit was used for the next steps including secondary antibody incubation, 3,3′‐diaminobenzidine staining, and hematoxylin staining. Finally, the slides were examined by routine light microscopy (DM6 M LIBS, Leica).

### 5.4 Western blotting

Tissues or cells were dissociated on ice in radio‐immunoprecipitation assay lysis buffer with protease inhibitor cocktail for 1 h. The homogenates were centrifuged at 15,000 ×*g* for 120 min at 4°C to separate the supernatants. The protein concentrations were detected by bicinchoninic acid protein assays and analysis kits. A total of 30 μg protein lysates were separated by 4%–12% NuPAGE Bis‐Tris gel electrophoresis by standard methods with the primary and secondary antibodies mentioned above. Finally, the bands were detected in iBright Gel imaging system (Thermo Fisher Scientific).

### 5.5 HE staining and Oil Red O staining

For HE staining, paraformaldehyde‐fixed liver tissue was embedded in paraffin and sliced into 6‐μm sections. The slides were baked at 60°C and dewaxed by dimethylbenzene. After alcohol treatment with an inverse concentration gradient, the slides were stained by HE with HE Stain Kit according to established protocols. Finally, the slides were examined by routine light microscopy (DM6 M LIBS, Leica).

For Oil red O staining, paraformaldehyde‐fixed tissues were embedded in optimal cutting temperature compound and sliced into 10‐μm sections. The cells were cultured in a Nunc Lab‐Tek Chamber Slide System (Thermo Fisher Scientific). The slides were treated with isopropanol for 5 min. Oil Red O staining, hematoxylin staining, and slide sealing were performed with an Oil Red O Stain Kit according to established protocols. Finally, the slides were examined by routine light microscopy (DM6 M LIBS, Leica).

### 5.6 ALT assay, AST assay, and TAG detection

For ALT and AST assays, mouse serum was extracted at 1000 ×*g* for 5 min at 4°C, and the supernatants were separated and treated with ALT or AST assay kits according to established protocols. Finally, the levels of ALT or AST were detected with a colorimeter (Cary 60 UV‒Vis, Agilent) at a wavelength of 505 nm.

For TAG detection, the liver tissues were dissociated on ice in isopropanol. The homogenates were centrifuged at 15,000 ×*g* for 120 min at 4°C, and the supernatants were collected and treated with a Triglyceride Detection Kit according to established protocols. Levels of TAG were detected with a colorimeter (Cary 60 UV‒Vis) at a wavelength of 505 nm.

### 5.7 Data collection, single‐cell RNA sequencing data processing, and GO enrichment

The RNA‐seq datasets of GSE185051 and GSE200482, as well as microarray dataset GSE200409, were obtained from the Gene Expression Omnibus (GEO) database with log_2_ transformation. The R (version 4.0.4) and RStudio (version 1.2.5033) were used to address all the data in this study. GSE185051 contained 52 NAFLD samples and five healthy liver samples, GSE200482 contained four normal chow diet C57BL6J mouse liver samples and five SSD C57BL6J mouse samples, and GSE200409 contained 12 mouse liver samples from mice fed a normal or HFD for different times.

scRNA‐seq data from the GSE166504 dataset were obtained from the GEO database.^14^ The R package “Seurat” (version 4.0.2) was used to process the data. Six hepatocyte samples from mice fed a HFHFD for 15 weeks and four hepatocyte samples from mice fed a HFHFD for 30 weeks were selected for analysis. The scRNA‐seq data were analyzed as we previously described.^43^ The t‐distributed stochastic neighbor embedding algorithm was used to explore and visualize cluster classifications across the cell samples. The cell clusters were annotated manually based on the related metadata of GSE166504.

The “clusterProfiler” (version 3.18.1) R package was used for GO enrichment analyses, which included biological process, molecular function, and cellular component. The *p*‐value was set at 0.05.

### 5.8 Transmission electron microscopy

The samples were treated 4% glutaraldehyde as we described previously.^12^, ^26^ The dissection and staining were performed by the Advanced Research Center, Central South University, China. The thickness of the section was 70 nm, and the staining was performed using uranyl acetate and lead citrate. The pictures were collected by TEM (H‐7650; Hitachi).

### 5.9 ATP assay and ROS assay

Liver tissues or cells were homogenized on ice with extraction solution. After centrifugation, the supernatants were collected for detection. The ATP levels were measured by phosphomolybdic acid colorimetry with an ATP array kit at a wavelength of 340 nm with a colorimeter (Cary 60 UV‒Vis). The ROS levels were measured by fluorometric analysis using 2,7‐dichlorofluorescein diacetate with an ROS array kit. The ROS levels were detected by colorimeter (Cary 60 UV‒Vis) with 488 nm excitation wavelength.

### 5.10 RNAi screening and plasmid transfection

The HT115 bacteria which included the plasmids that can express the dsRNAs to target the ret‐1 and silence the expression of ret‐1 were prepared for adult *C. elegans*.

The WT RTN3 oding sequence (CDS) with a C‐terminal Flag‐tag in pcDNA3.1+ was designed and structured. The L02 cells were transiently transfected with pcDNA3.1‐blank and pcDNA3.1‐RTN3 using Lipofectamine™ 3000 CD Transfection Reagent following the manufacturer's instructions.

### 5.11 RNA‐seq and RT‐PCR

Total RNA was isolated from tissues or cells with an RNA isolation kit. The RNA‐seq and bioinformatics analyses were performed by the BerryGenomics Biotech company (Beijing, China). cDNA was synthesized by RevertAid First Strand cDNA Synthesis Kit with 1 μg RNA and prepared with Maxima SYBR Green/ROX qPCR Master Mix (2×). Finally, the Fast 7500 RT‐PCR Systems (Applied Biosystems) and 2^(−△△Ct)^ methods were used to compare the RNA levels of each group.

### 5.12 MS analysis and Co‐IP

The protein supernatants of WT mouse liver tissues were extracted as described in the WB methods. A total of 500 μg lysates in 1 mL were used for MS and Co‐IP with primary antibody (IgG or anti‐RTN3) and Protein A + G beads overnight. After denaturation by heating and centrifugation, the supernatants were separated by 4%–12% NuPAGE Bis‐Tris gel electrophoresis. For MS analysis, the gel was stained with Coomassie blue staining solution R250 (ST1123, Beyotime Biotechnology), and the potential bands were cut and sent to Novogene Bioinformatics Institute (Beijing, China) for further MS analysis. For Co‐IP, the supernatants were detected by standard WB methods with antibodies against RTN3 and GRP78. The bands were detected in iBright Gel imaging system (Thermo Fisher Scientific).

### 5.13 Statistical analysis

The data were subjected to statistical analysis with GraphPad Prism 6 (GraphPad Software). Five independent repetitions were conducted for each group in this study. Image J was used to perform the WB grayscale analysis. All data are presented as means ± standard deviation. Data were analyzed using paired Student's *t*‐test. *p* < 0.05 was considered significant.

## AUTHOR CONTRIBUTIONS

H.H. and S.G. wrote the draft of the manuscript and performed the bioinformatic analysis and cell and molecular experiments. Y.‐Q.C. enrolled the patient's samples. Y.‐X.L. and J.‐Y.J. performed hematoxylin–eosin and immunohistochemistry. Y.L. performed animal feeding. L.‐L.F. and R.X. revised the manuscript and designed and supported the project. All authors approved the final manuscript.

## CONFLICT OF INTEREST STATEMENT

The authors declare no conflicts of interest.

## ETHICS STATEMENT

All procedures followed were in accordance with the ethical standards of the Helsinki Declaration of 1975, as revised in 2000. This study was approved by the Institutional Review Board committee of the Central South University in China (approval number: No. 2021‐1‐1 for human specimen, No. 2021‐2‐1 for animal, date: February 24, 2021). All patients provided written informed consent.

## Supporting information

Supporting Information

## Data Availability

Experimental data related to the article are available from the corresponding author.
