# Correction to: Increased RTN3 phenocopies nonalcoholic fatty liver disease by inhibiting the AMPK‐IDH2 pathway

**DOI:** 10.1002/mco2.466

**Published:** 2024-02-04

**Authors:** Hao Huang, Shuai Guo, Ya‐Qin Chen, Yu‐Xing Liu, Jie‐Yuan Jin, Yun Liang, Liang‐Liang Fan, Rong Xiang

**Affiliations:** ^1^ Department of Nephrology National Clinical Research Center for Geriatric Disorders Xiangya Hospital, Central South University Changsha China; ^2^ Department of Cell Biology School of Life Sciences Central South University Changsha China; ^3^ Hunan Key Laboratory of Animal Models for Human Diseases School of Life Sciences Central South University Changsha China; ^4^ National Clinical Research Center for Geriatric Disorders Xiangya Hospital Central South University Changsha China; ^5^ Department of Cardiovascular Medicine the Second Xiangya Hospital Central South University Changsha China

In the process of checking the raw data,^1^ the authors noticed an inadvertent mistake occurring in Figure 1F that needed to be corrected after the online publication of the article. During the preparation of Figure 1F, the representative image showing the expression of RTN3 was pasted and placed by mistake. The correct result should be as shown below. The authors apologize for these oversights and declare that this correction does not affect the description, interpretation, or conclusions detailed in the original manuscript.

**FIGURE 1:**
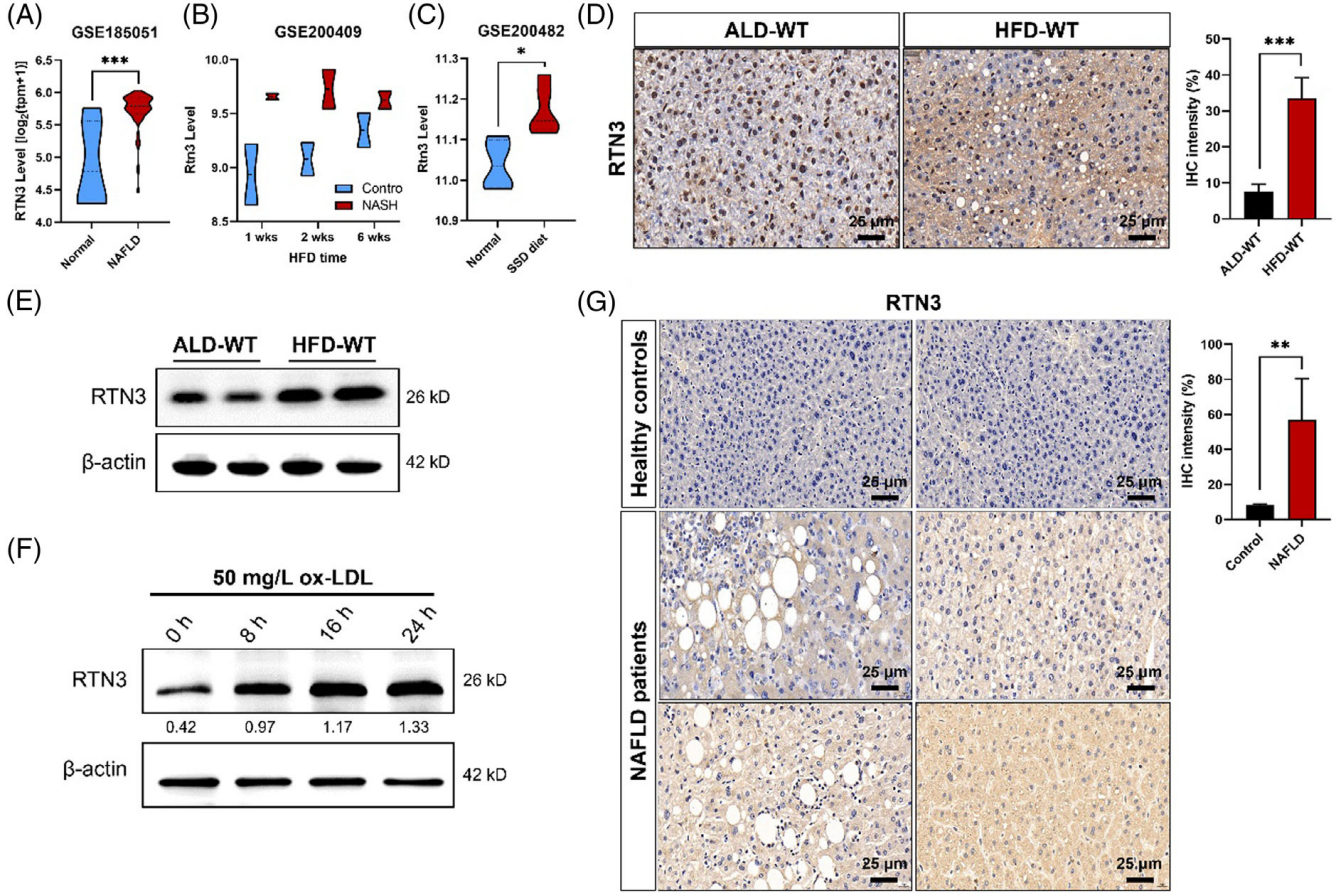
Link between high expression of reticulon 3 (RTN3) and nonalcoholic fatty liver disease (NAFLD). The mRNA levels of RTN3 in NAFLD patients and healthy control (A), in high‐fat diet (HFD) mice and wild‐type (WT) control (B), and in steatosis‐steatohepatitis diet (SSD) mice and WT control (C). The protein levels of RTN3 were detected in ad libitum diet (ALD)‐WT and HFD‐WT mice liver tissues by immunohistochemistry (IHC) (D) and western blotting (WB) (E). (F) WB showed the RTN3 levels in oxidized low‐density lipoprotein (ox‐LDL) (50 mg/L) treated L02 cell lines in different time. (G) IHC showed the RTN3 levels in liver tissues of healthy controls and NAFLD patients. *p < 0.05, **p < 0.01, and ***p < 0.001. NASH, nonalcoholic steatohepatitis.

## References

[1] Huang H , Guo S , Chen YQ , et al. Increased RTN3 phenocopies nonalcoholic fatty liver disease by inhibiting the AMPK‐IDH2 pathway. MedComm. 2023;4(2):e226. doi:10.1002/mco2.226 36925557 PMC10013133

