# Supplementary material for: Increased RTN3 phenocopies nonalcoholic fatty liver disease by inhibiting the AMPK–IDH2 pathway
Source: MedComm (2020). 2023 Mar 14;4(2):e226. doi: 10.1002/mco2.226 (PMC10013133; doi:10.1002/mco2.226)
Supplement: Supplementary file 1 — Supporting Information [file MCO2-4-e226-s001.docx]

**Increased RTN3 phenocopies nonalcoholic fatty liver disease by inhibiting the AMPK-IDH2 pathway**

**Running title:** Increased RTN3 leads to NAFLD.

Hao Huang^1, 2, 3, 4#^, Shuai Guo^2#^, Ya-Qin Chen^5^, Yu-Xing Liu^2^, Jie-Yuan Jin^2^, Yun Liang^2^, Liang-Liang Fan^2,3*^, Rong Xiang^1,2,3,4,5*^

1. Department of Nephrology, National Clinical Research Center for Geriatric Disorders, Xiangya Hospital, Central South University, Changsha, 410008, China;

2. Department of Cell Biology, School of Life Sciences, Central South University, Changsha, 410013, China;

3. Hunan Key Laboratory of Animal Models for Human Diseases, School of Life Sciences, Central South University, Changsha, 410013, China;

4. National Clinical Research Center for Geriatric Disorders, Xiangya Hospital, Central South University, Changsha, 410008, China.

5. Department of Cardiovascular Medicine, the Second Xiangya Hospital, Central South University, Changsha, 410010, China.

# Contributed equally.

* Correspondence:

Liang-Liang Fan, Ph.D

Email: [swfanliangliang@csu.edu.cn](mailto:swfanliangliang@csu.edu.cn)

and

Rong Xiang, Ph.D.

Email: [shirlesmile@csu.edu.cn](mailto:shirlesmile@csu.edu.cn)

Department of Cell Biology, The School of Life Sciences, Central South University, Changsha 410013, China.

Table S1. Clinical and biochemical characteristics of NAFLD patients

| Participants ID | Age | Gender | BMI  (kg/m2) | Triglyceride  (mmol/L) | Cholesterol  (mmol/L) | HDL-LDL  (ratio) |
| --- | --- | --- | --- | --- | --- | --- |
| Patient 1 | 35 | Male | 31.4 | 6.05 | 4.74 | 3.28 |
| Patient 2 | 42 | Male | 26.7 | 4.19 | 4.03 | 3.35 |
| Patient 3 | 37 | Female | 33.9 | 7.07 | 5.09 | 3.17 |
| Patient 4 | 43 | Male | 26.3 | 4.54 | 4.32 | 2.89 |
| Control 1 | 39 | Male | 23.8 | 1.48 | 3.43 | 2.42 |
| Control 2 | 35 | Male | 24.3 | 1.32 | 4.06 | 2.69 |

Table S2. The key reagents in the study.

| **REAGENT or RESOURCE** | **SOURCE** | **IDENTIFIER** |
| --- | --- | --- |
| **Antibodies** | | |
| MFN2 Polyclonal antibody | Proteintech | 12186-1-AP |
| FIS1 Polyclonal antibody | Proteintech | 10956-1-AP |
| OPA1 Antibody (D-9) | Santa Cruz Biotechnology | sc-393296 |
| GRP78 Antibody (76-E6) | Santa Cruz Biotechnology | sc-13539 |
| AMPK alpha 1 Antibody (H-4) | Santa Cruz Biotechnology | sc-398861 |
| Phospho-AMPKα (Thr172) (40H9) Rabbit mAb | Cell Signaling Technology | #2325 |
| IDH2 (D8E3B) Rabbit mAb | Cell Signaling Technology | #56439 |
| β-Actin (13E5) Rabbit mAb | Cell Signaling Technology | #4970 |
| Anti-rabbit IgG, HRP-linked Antibody | Cell Signaling Technology | #7074 |
| Anti-mouse IgG, HRP-linked Antibody | Cell Signaling Technology | #7076 |
| **Chemicals, Reagents and Kits** | | |
| Pierce™ BCA Protein Assay Kit | Thermo Fisher Scientific | 23227 |
| PureLink® RNA Mini Kit | Thermo Fisher Scientific | 12183025 |
| Lipofectamine™ 3000 | Thermo Fisher Scientific | L3000150 |
| RevertAid First Strand cDNA Synthesis Kit | Thermo Fisher Scientific | K1621 |
| Maxima SYBR Green/ROX qPCR Master Mix (2×) | Thermo Fisher Scientific | K0221 |
| Fetal Bovine Serum, qualified, heat inactivated, Australia | Thermo Fisher Scientific | 10100147 |
| PageRuler™ Plus Prestained Protein Ladder, 10 to 250 kDa | Thermo Fisher Scientific | 26620 |
| RIPA Lysis and Extraction Buffer | Thermo Fisher Scientific | 89901 |
| NuPAGE™ 12%, Bis-Tris, 1.0 mm, Mini Protein Gels | Thermo Fisher Scientific | NP0341BOX |
| Ox-LDL | Solarbio Life Sciences | H7950 |
| Broad Spectrum Immunohistochemistry Kit | Solarbio Life Sciences | SP0041 |
| Hematoxylin-Eosin/HE Stain Kit | Solarbio Life Sciences | G1120 |
| Oil Red O Stain Kit | Solarbio Life Sciences | G1261 |
| Alanine transaminase (ALT) assay Kit | Solarbio Life Sciences | BC1550 |
| Aspartate transaminase (AST) assay Kit | Solarbio Life Sciences | BC1560 |
| Adenosine triphosphate (ATP) assay Kit | Solarbio Life Sciences | BC0300 |
| Reactive Oxygen Species (ROS) Assay Kit | Solarbio Life Sciences | CA1410 |
| OCT Compound | Solarbio Life Sciences | 4583 |
| Penicillin-Streptomycin Liquid (100×) | Solarbio Life Sciences | P1400 |
| Triglyceride detection kit | Nanjing Jiancheng Bioengineering Institute | A110-1 |
| 100× Protease Inhibitor Cocktail | Sangon Biotech | C600386 |
| Protein A+G beads | Beyotime Biotechnology | P2197S |
| Coomassie blue staining solution R250 | Beyotime Biotechnology | ST1123 |
| Dulbecco’s Modified Eagle’s Medium (DMEM) with high glucose | Procell Life Science&Technology | PM150210 |
